# State Education

**Published:** 1882-07

**Authors:** Theophrastus Schopenhauer


### STATE EDUCATION.

THEOPHRASTUS SCHOPENHAUER.

II.

My introductory article developed severe postulates of a two-fold character—positive and negative.

Much fruitful and animated discussion and no little additional reading, as well, have failed to discover any radical divergence of opinion as to the former, between the champions of what may for brevity's sake be termed aptly enough "text-book" and "Quincy" teaching, respectively. These propositions, therefore, may be re-stated as incontrovertible:

First—train the *senses* (perceptive faculties.)

Next—drill the *memory* (retentive faculties.)

Last—school the *mind* (reasoning faculties.)

But controversy does wage over the following proposition; nevertheless I boldly reaffirm that

Ninety-nine hundredths of our children might as well be born *without* their seven senses—so little is done at home, no less than in the schools to utilize the same for the purpose of acquiring a knowledge of things material; that

It is essentially the memory alone which is developed in the Primary school, while in the "Grammar" school, socalled, little else is accomplished beyond *littering* the mind with abstract or alleged facts; and that

The endeavors of most High Schools are thus of necessity restricted to "*disciplining*" the mind, instead of equiping it with the ability clearly, to understand isolated or co-related facts and to reason from them rapidly and correctly.

Now, it goes without saying that it would be no less unfair than impolitic not to admit that these assertions are *in some sense* an exaggeration of the real state of affairs, unwarrantable, except for the purposes of dogmatic emphasis. But that they *do*, rather vividly if you please, yet none the less truthfully, describe the actual results of the American Common School System of which as a nation, we are despite all *justly* so proud—a casual yet thoughtful enumeration of certain undeniable facts, will amply serve to demonstrate.

This is no agreeable task, viewed either subjectvely or objectively—for as the profession well knows: the state of affairs technically denominated "*odium pædagogicum*" is quite as sure to engender virulent and protracted wrangling, to no purpose, as is its famous counterpart among the theologians; and, moreover, I am well aware that such a course will be considered by not a few of my misguided, but none the less highly esteemed fellow-citizens, either as "characteristically Quixotic" or as "just too conceited and personal for anything!" according to their sex or previous condition of *con*-servitude, respectively.

How be it—to my fellow-teachers and educators generally I need not commend the sense and spirit of that admirable French proverb: "*Discutons toujours; Disputons jamais;*" and as to irrelevant or illogical assaults from other quarters, can they serve any other purpose than that of supplying the "Q. E. D." to my argument?

I am not, of course, prepared to make the assertion dogmatically, in view of the "S. T.—1860—X." of other days, or the undeniable virtues of "St. Jacob's Oil," whose uses are daily becoming more obvious and manifold, as it were—but it has always seemed to me as though "Radway's Ready Relief" must be calculated to relieve the inner no less than the outer man and beast more thoroughly of their ills than any other product of this enlightened age and liberty-loving people—inasmuch as it is quite as firmly rooted and grounded in "*the three R's*" as is our Public School System itself! Let us nevertheless subject them to some little critical examination.

Prefatory to nearly every text-book of "Grammar," so-called, we find the statement: "*English Grammar is the Art of Speaking, Reading and Writing the English Language correctly;*" and we all know that some six years or more are conscientiously devoted to indoctrinating the "rules" of this "Art" such wise, that our boys and girls can "analyze" with such celerity as to take a fellow's breath quite away, and "diagram" with so much color and picturesqueness as to engender envy in the breast of a Wyatt Eaton; but, upon "graduating," are they able to speak their mother tongue correctly, to read it intelligibly (not to say intelligently) or to write it fluently? I could tell of a bright, studious and ambitious child who "passed" her "Regents" in Grammar with a percentage of *ninety-nine and a fraction*, "standing at the *head*" of her class—whose accent and idiom, however, remain to-day as deliciously Teutonic as ever they were before she "entered" school; and is there any one to dispute that similar results obtain, *mutati mutandis*, where the scholar's vernacular has at home been tinged either with Milesian brogue or Yankee twang? What accomplishment (*sic!*) so rare as that of transcribing events exactly and vividly, or of projecting thought lucidly and tersely? And is it not a matter of common observation that of men and women, grown even, not one in twenty is able to read half a dozen ordinary sentences at sight without mispronunciation, false inflection, halting, repetition and confusion of all sorts in plenty?

And as to 'rithmetic—do our scholars fare any better? The text-books "take them through" (*sic!* again) Quadratics "up to" Stock Gambling and Insurance—an Appendix on the Mutual, Matrimonial and Grave-yard varieties of the latter, to be supplied by the enterprising publishers in the very next edition, I have no doubt, so as to keep the "series" abreast of the times. The upshot is more or less dexterity at "figuring" and no end of rule-rattling; only this and nothing more. Let him of contrary opinion ask his boy what shall be the dimensions of the feed bins in the new barn that are to hold respectively seventy-five bushels of oats and thirty of corn, or hand him a note endorsed with a number of part payments, and demand a statement of principal and interest still due; let his wife request the "sweet girl graduate" to make a plum pudding just for "Ma," "Pa," "Tom" and herself, reduced from the Thanksgiving recipe's more ample proportions—or to "cypher out" how many yards it would take to furnish the windows with curtains, the mantle shelf with a *lambrequin* and the centre-table with a spread—and who doubts the result?

These facts granted, since no one will dispute them, we are, however, immediately met with the assertion, intended as an offset: "Nevertheless, the youthful mind is disciplined, the memory is strengthened."

This achievement we should be the last to undervalue, *were* it accomplished even at the sacrifice of a correct and thorough *knowledge* of our mother-tongue, and of a ready, practical *understanding* of the respective functions and attributes of number and quantity—their corelation and interdependence. But I am constrained to deny this assumption; and such familiarity as I possess with the maturest and fairest thought that literature affords in the domain of theoretical and applied pædagogy, fully warrants the assertion that the rarest phenomenon encountered by High School teacher and College professor is a good—I am almost tempted to say a *fair*—memory; frank discussion with many teachers strengthens this belief, and repeated, careful review of my school experience of twenty years, both as scholar and teacher, only serves to confirm the same. *So far from strengthening the memory, rote-learning in the end actually ab-absorbs it!* just as the protracted use of narcotics slowly but surely deprives them of all their efficacy.

*Nor, indeed, does the text-book "discipline" the mind.* Its condensed, cast-iron dogmatism applies formulas in place of facts, and substitutes the catching jingle of ready-made definitions for the things themselves. Thus: if one should happen to remark, by way of explanation, that the Indicative Mood is employed in the phrase, "If it rains," since the verb is intended to express a future action as sure to come about, he would instantly and incontinently be abashed by the statement: "*That can't be so, for* THE "GRAMMAR SAYS *that* 'if is the sign of the "subjunctive.'" Or again: were one to make inquiry as to the addition of fractions, for instance, there would be no lack of "rules," but the reason—nowhere.

"Discipline," forsooth—when each new fact baffles the intellect by its absolute uniqueness and startling novelty. "Memory," indeed—when everything that is not "committed" is forgotten!

